# Water Sorption and Solubility of Highly Aesthetic Single-Shade Nano-Hybrid Resin Composites

**DOI:** 10.7759/cureus.65548

**Published:** 2024-07-27

**Authors:** Sumayyah L Alkhudhayri, Shahad L Alhassani, Reem J Alahmadi, Mariam R Alharbi, Hetaf S Redwan, Rayan A Hawsawi

**Affiliations:** 1 College of Dental Medicine, Umm Al-Qura University, Makkah, SAU; 2 Restorative Department, College of Dental Medicine, Umm Al-Qura University, Makkah, SAU

**Keywords:** surface roughness, single shade composite, nanohybrid composite, water solubility, water sorption

## Abstract

Background

A single-shade resin composite is a new type of resin composite that was introduced in 2019. There is not much data regarding the water sorption (Wsp) and water solubility (Wsl) of this type of resin composite. Therefore, this study aimed to investigate Wsp and Wsl and their effects on the surface roughness of a single-shade nano-hybrid resin composite in comparison with a conventional nano-hybrid resin composite in accordance with ISO 4049:2019 (Dentistry - Polymer-based Restorative Materials).

Material and methods

An in vitro study was performed to investigate the Wsp and Wsl of a single-shade supra-nano-hybrid composite (Omnichroma) and a conventional nano-hybrid composite (Filtek^™ ^Z250 XT) in accordance with ISO 4049:2019. Five disks were prepared of each material with dimensions of 15 ± 1 mm in diameter and 1 ± 0.1 mm in thickness, as per ISO 4049:2019. The results were calculated according to the ISO equations for Wsp and Wsl in µg/mm^3^. Atomic force microscopy (AFM) was used to analyze the surface roughness (R_a_) of test specimens.

Results

The findings showed that the values of Wsl and Wsp of both materials are comparable and revealed no statistically significant difference (P > 0.05). The AFM images showed a higher R_a_ post-solubility testing, and the statistical analysis indicated a significant difference for both materials.

Conclusions

The study concluded that the single-shade resin composite (Omnichroma) and the conventional nano-hybrid composite (Filtek^™ ^Z250 XT) are following the requirements of ISO 4049:2019. The AFM analysis indicated that resin composite surfaces are significantly affected when exposed to water for a prolonged period of time. However, the R_a_ variations of Filtek^™ ^Z250 XT were higher than Omnichroma specimens. These results indicate that resin composite surfaces can be significantly impacted by prolonged water exposure. This knowledge is critical for enhancing the long-term clinical performance and durability of these dental restorative materials.

## Introduction

The conservative approach in dentistry aims to increase the longevity of the restoration and preserve the tooth structure, so new promising materials were introduced to be applied in this approach [1]. Resin composite materials provide a better aesthetic with a minimal amount of tooth structure removal [2]. It has been found that there are some drawbacks of conventional composites (macro-filled resin composites), such as water sorption (Wsp), which is the process of influx of water into the composite matrix due to changes in the oral temperature, filler types in restoration, and matrix/filler interference in restoration [3,4]. Microcracks can be created due to the hydrolytic degradation of Wsp by breaking bonds between the resin matrix and fillers [4,5]. A study claimed that many types of composites showed a significant difference in hygroscopic expansion when exposed to water [6]. Another study found that the Wsp of low-shrinkage resin composites and conventional resin composites increased in all tested composite resins after a year of storage in water, but there were no changes in water solubility (Wsl) [7].

In addition, researchers have found that Wsl influences the surface hardness, which is a pre-indicator for the wear resistance of composite restorations, and leads to chemical softening that may affect the longevity of composite restorations [8,9]. It has been found that the fillers’ matrix and chemical composition of resin composites have a hydrophilic effect that may change the sensitivity of Wsp and Wsl to time and pH [10]. The solubility of resin-based composite materials can decrease their wear and abrasion resistance, as well as their color stability [11]. The popularity and reliability of composite resin restorations have grown significantly. Classification of resin composites based on particle size is the most commonly used way, which divides composites into macroparticle hybrids, micro-hybrids, microparticles, nanoparticles, and nano-hybrids. This development aimed to improve the mechanical and aesthetic properties as well as minimize polymerization shrinkage [12].

A new restorative composite material (Omnichroma) was introduced in 2019, which is known as a shade-matching composite. It is claimed that this material can match any tooth color and shows superior aesthetic properties compared to conventional composites [13]. Moreover, some studies have demonstrated that this universally highly aesthetic material has proper mechanical and wear resistance properties [14]. It appears that the interest in the longevity of resin composite restorations is not only confined to good mechanical and optical properties, but it also includes acceptable chemical properties by obtaining data on Wsp and Wsl. It is therefore important to understand the Wsp and Wsl of resin-based composite materials and their effects to properly determine the mechanical, physical, and biological properties of the restorations [3]. The lack of data indicates the significance of measuring the Wsp and Wsl and their effects on this newly introduced type of composite (single-shade composite). Therefore, this study aimed to investigate Wsp and Wsl and their effects on the surface roughness of a single-shade nano-hybrid resin composite in comparison with a conventional nano-hybrid resin composite in accordance with ISO 4049:2019 (Dentistry - Polymer-based Restorative Materials). The null hypothesis was that there is no difference between single-shade composites and conventional composites regarding the findings of Wsp and Wsl.

## Materials and methods

**Figure 1:**
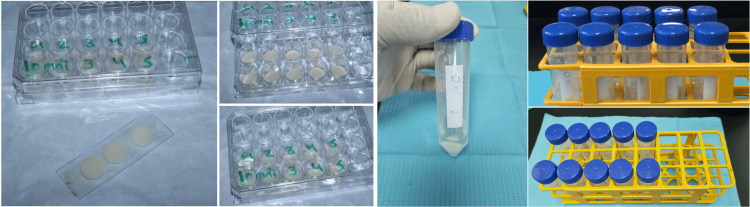
Five specimens of a single-shade nano-hybrid composite (Omnichroma) and a conventional nano-hybrid composite (Filtek™ Z250 XT) were prepared according to ISO 4049:2019 standards. The specimens were marked and carefully handled. Each specimen was immersed in water in a separate container at 37 ± 2 °C in an oven for one week, maintaining a vertical position.

A single-shade nano-hybrid composite (Omnichroma) (Tokuyama, Japan) and a conventional nano-hybrid composite (Filtek™ Z250 XT) (3M ESPE, United States) were used in this study. The experiment requires five specimens of each material as per ISO 4049:2019, as shown in Figure 1. A mold was prepared with dimensions of 15 ± 1 mm in diameter and 1 ± 0.1 mm deep, which was used to prepare five disks of each material as per ISO 4049:2019. The area (mm^2^) and the volume (mm^3^) were calculated.

According to ISO 4049:2019, the test specimens were placed in a desiccator at 37 ± 2 °C. After 22 hours, the specimens were removed and transferred to a second desiccator at 23 ± 1 °C for two hours. The specimens were weighed to an accuracy of 1.0 mg.

The cycle was repeated until a constant mass of m1 was reached, and there was no more than 0.1 mg of mass loss within a 24-hour period. The specimens were immersed in water at 37 ± 2 °C in an oven for one week and remained vertical, and it was important to keep at least 3 mm of space between specimens. Afterward, the specimens were removed and washed with water, blotted away surface water, and waved for 15 seconds in the air to keep the surface dry and free from visible moisture. After one minute, the weight was measured, and the mass was recorded as m2. In the desiccators, the cycle described earlier was performed to recondition the specimens to a constant mass, recorded as m3. Wsp was determined by calculating the added mass in µg/mm^3^: Wsp = m2-m3/V. Wsl was measured by calculating the mass loss in µg/mm^3^: Wsl = m1-m3/V.

Atomic force microscopy (AFM) (Veeco Instruments Nano-scope, United States) was used to analyze the surface roughness of Omnichroma and Filtek™ Z250 XT. A quantitative surface roughness analysis was performed by taking two measurements of all the study specimens: Ra1: pre-testing and Ra2: post-testing measurements for comparison. AFM analysis was performed on all specimens by scanning in contact mode. An independent t-test was performed to compare the average Wsp and Wsl values of Omnichroma and Filtek™ Z250 XT specimens and to compare the surface roughness measurements of both materials.

## Results

Wsp

**Figure 2:**
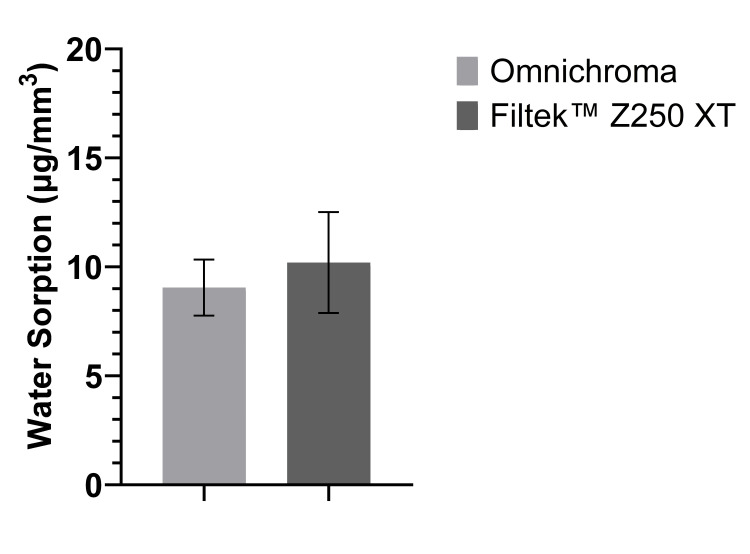
The mean of Wsp of Omnichroma specimens and Filtek™ Z250 XT specimens as per ISO 4049:2019 (bars represent standard deviation) (n = 5). Wsp, water sorption

Figure 2 shows that the mean Wsp of Omnichroma was 9.05 µg/mm^3^, which is less than the mean Wsp of Filtek™ Z250 XT (10.18 µg/mm^3^). For the Wsp comparison between the means of both materials, an independent t-test showed that the difference was not statistically significant (P > 0.05), as shown in Table 1.

**Table 1:** Wsl and Wsp values of Omnichroma and Filtek specimens using ISO 4049:2019 equations. * P < 0.05 was accepted as a significance level. * An independent t-test was performed. FL, Filtek; OM, Omnichroma; Wsl, water solubility; Wsp, water sorption

Specimen	Wsl (µg/mm^3^)	*P-value	Wsp (µg/mm^3^)	*P-value
OM1	11.31768	0.4937	-5.65884	0.8535
OM2	5.658842		-22.6354	
OM3	0		-22.6354	
OM4	11.31768		-5.65884	
OM5	16.97653		-5.65884	
FL1	22.63537		-5.65884	
FL2	0		-16.9765	
FL3	22.63537		-5.65884	
FL4	5.658842		-5.65884	
FL5	0		-11.3177	

Wsl

**Figure 3:**
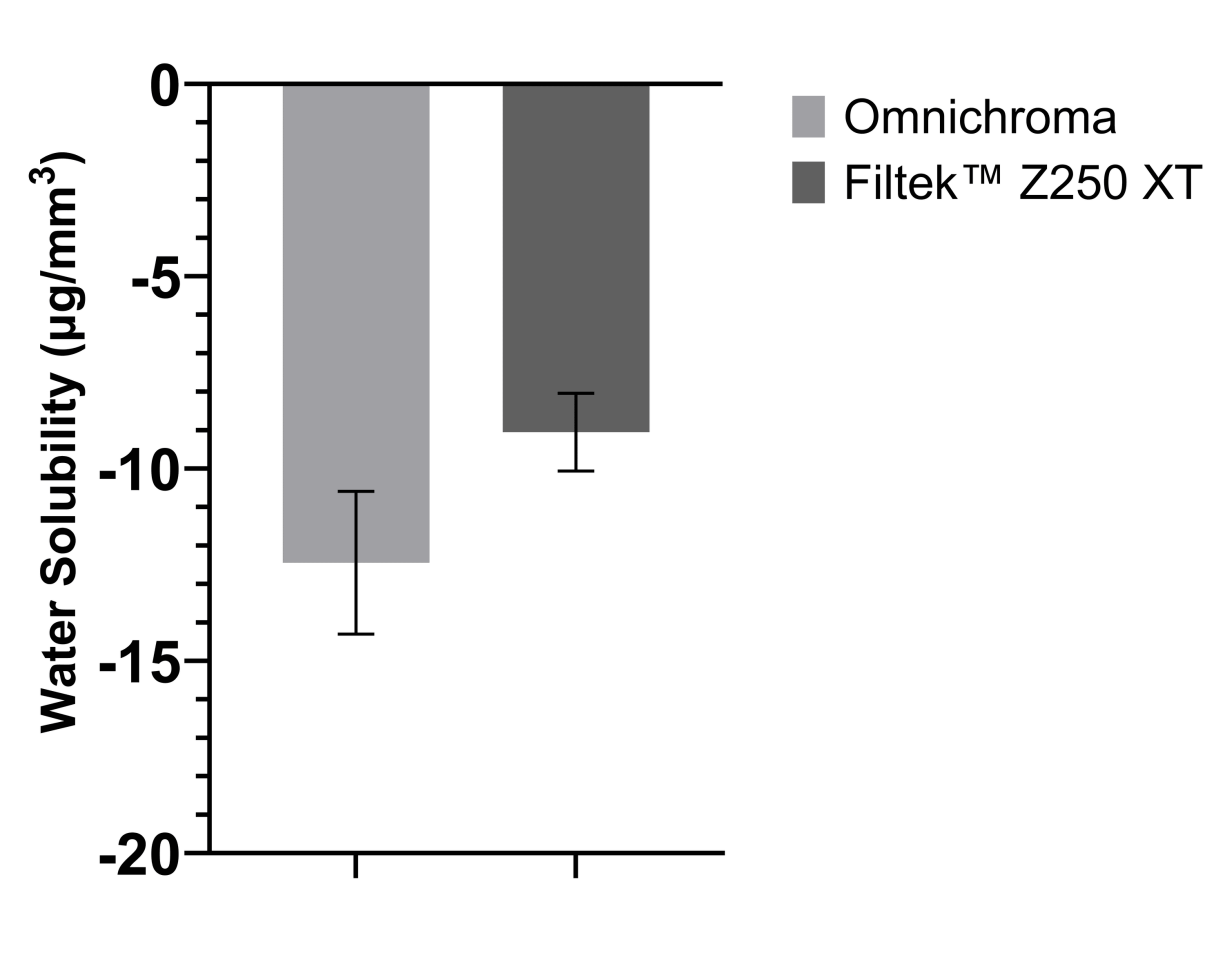
The mean of Wsl of Omnichroma specimens and Filtek™ Z250 XT specimens as per ISO 4049:2019 (bars represent standard deviation) (n = 5). Wsl, water solubility

Figure 3 shows that the mean of Wsl of Omnichroma was -12.44 µg/mm^3^, while the mean of Wsl of Filtek™ Z250 XT was -9.05 µg/mm^3^. For the Wsl comparison between the means of both materials, an independent t-test showed that the difference was not statistically significant (P > 0.05), as shown in Table 1.

Surface roughness

**Figure 4:**
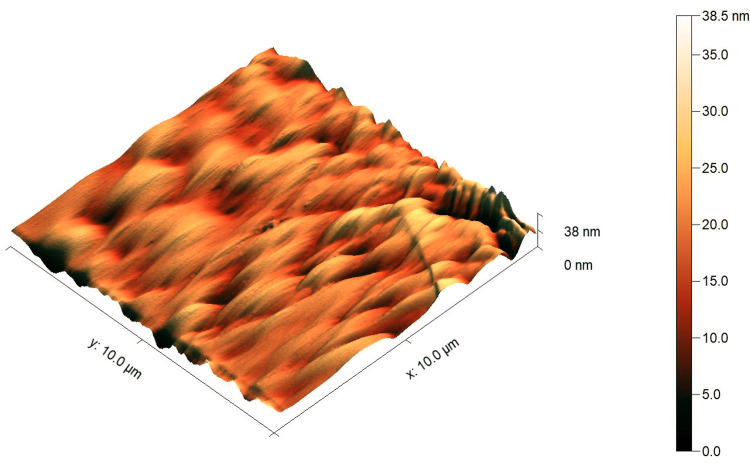
AFM images of Omnichroma specimens pre-testing. The right color bar shows the scale for the height in the image. AFM, atomic force microscopy

**Figure 5:**
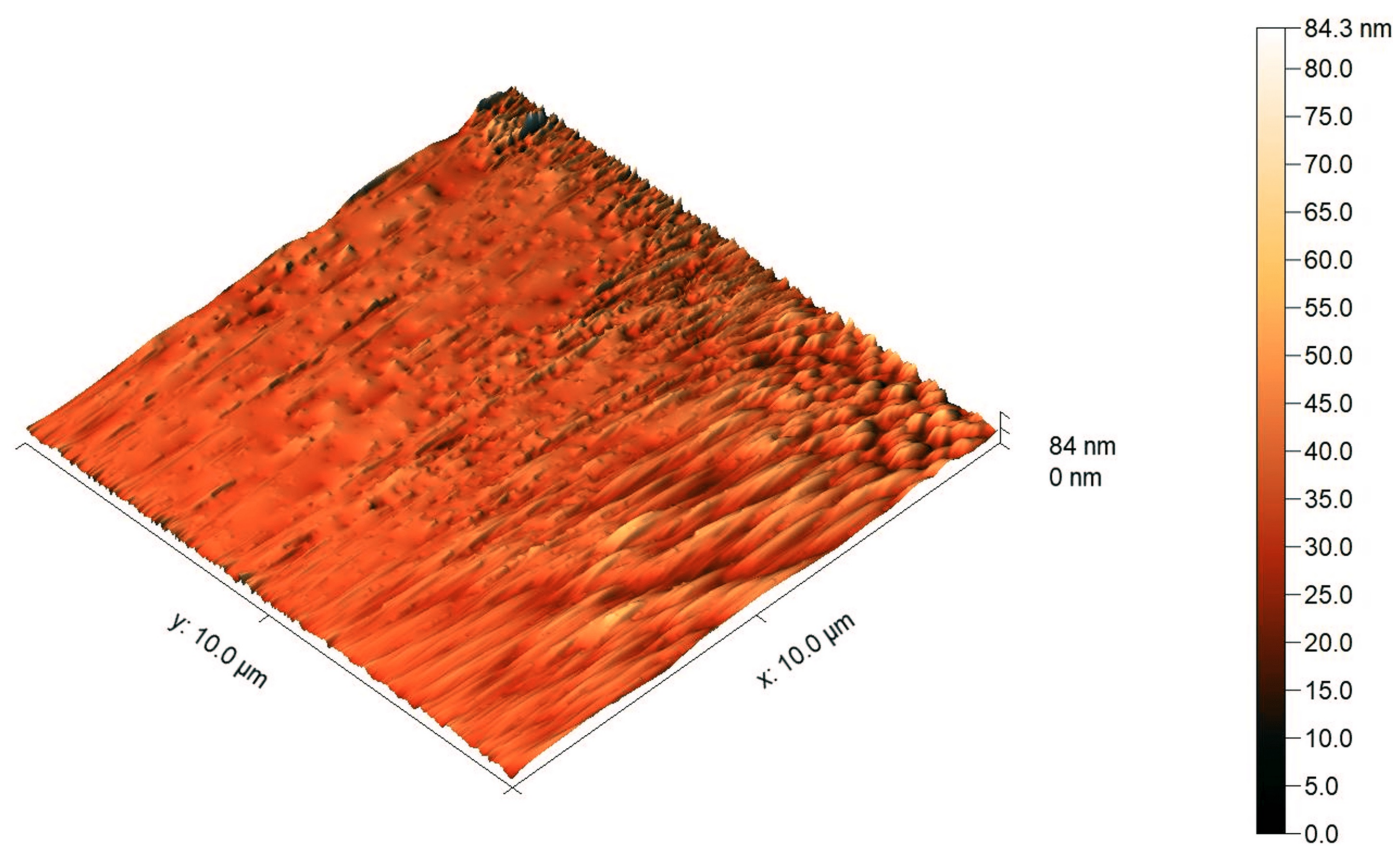
AFM images of Omnichroma specimens post-testing. The right color bar shows the scale for the height in the image. AFM, atomic force microscopy

**Figure 6:**
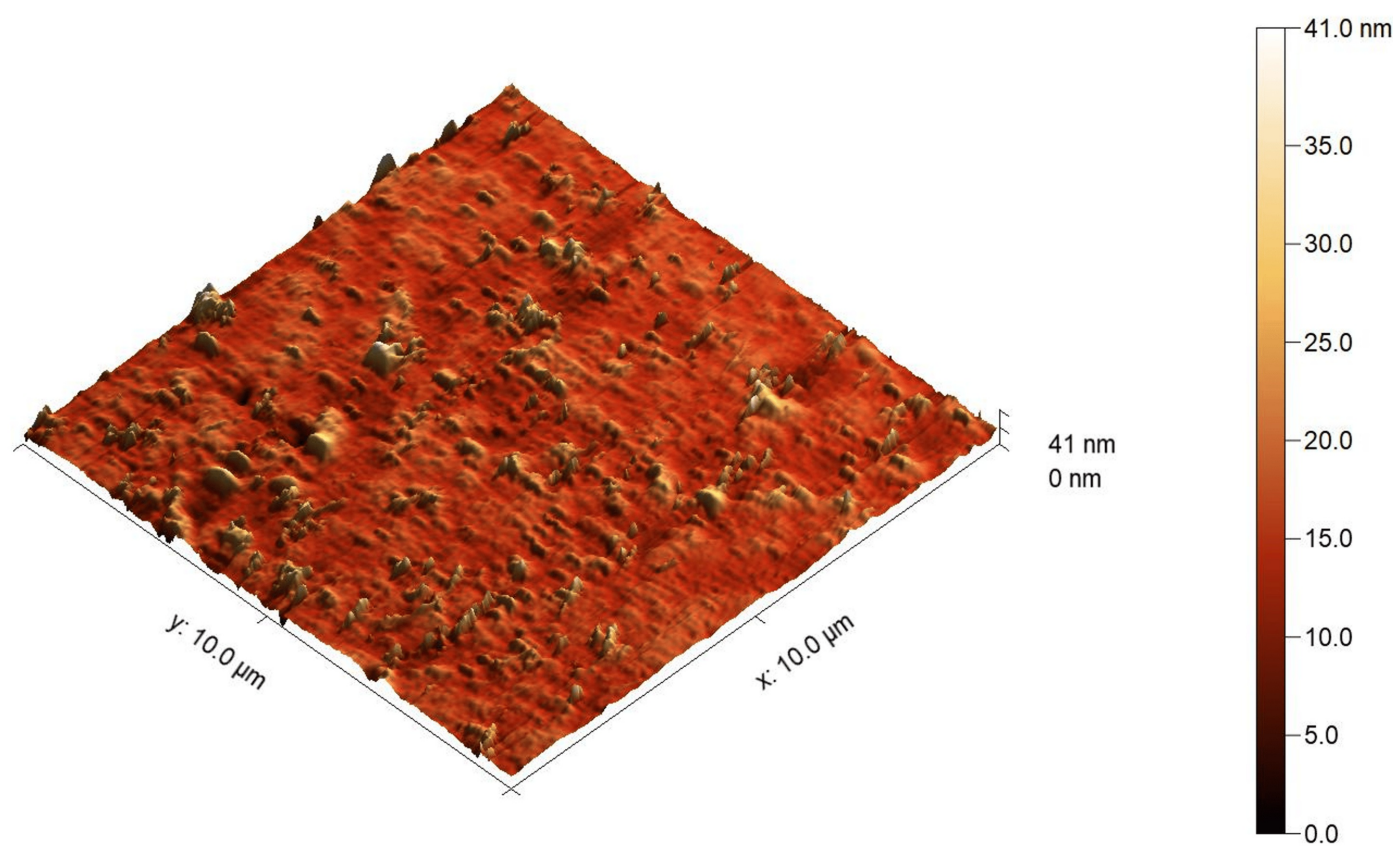
AFM images of Filtek™ Z250 XT specimens pre-testing. The right color bar shows the scale for the height in the image. AFM, atomic force microscopy

**Figure 7:**
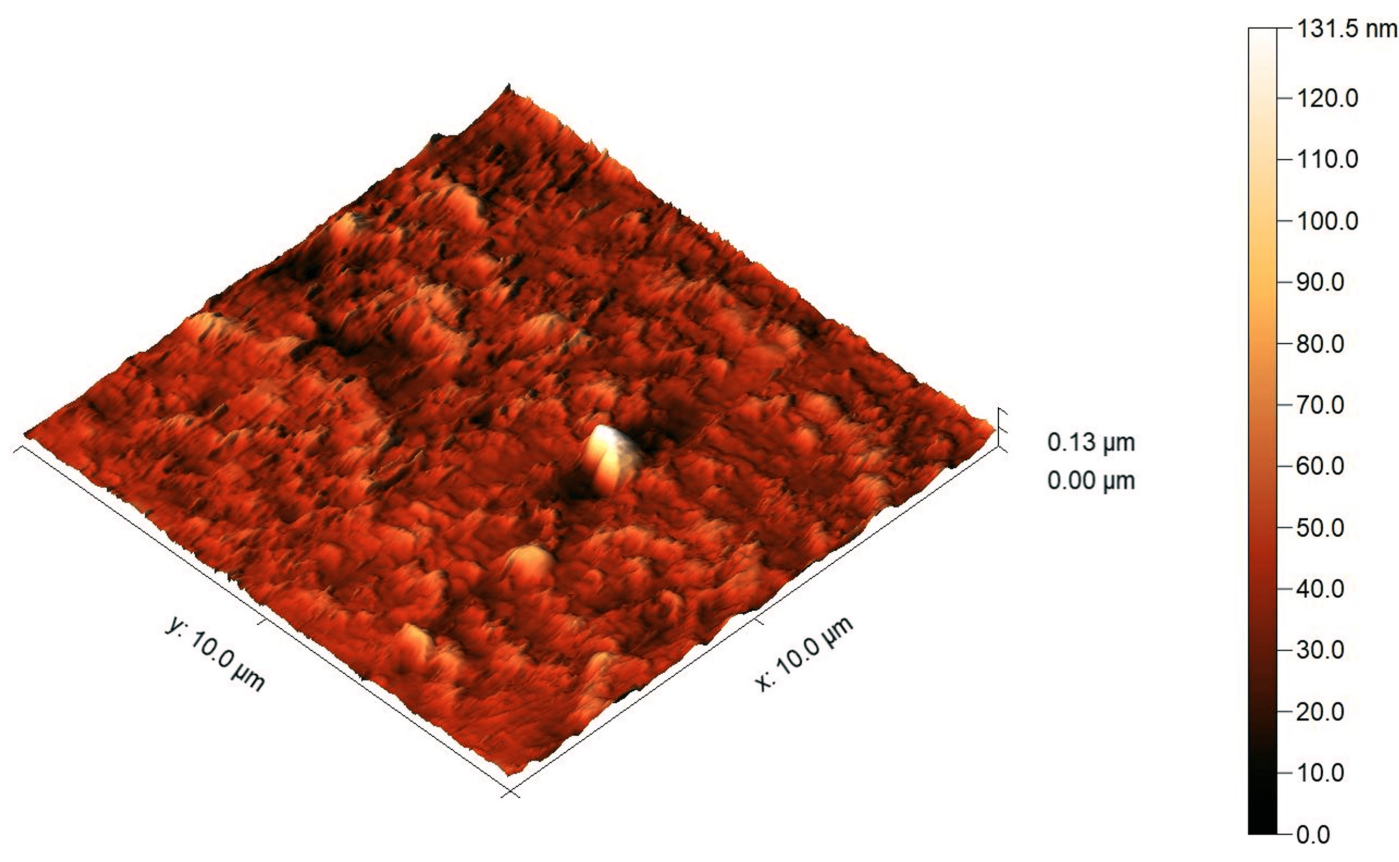
AFM images of Filtek™ Z250 XT specimens post-testing. The right color bar shows the scale for the height in the image. AFM, atomic force microscopy

The AFM images showed a higher surface roughness post-testing compared to pre-testing for both tested materials, as shown in Figure 4, Figure 5, Figure 6, and Figure 7. For Omnichroma specimens, it was observed that the maximum surface topography height increased from 38 nm to 84.3 nm post-testing, as shown in Figure 4 and Figure 5. For Filtek™ Z250 XT specimens, it was observed that the maximum surface topography height increased from 41 nm to 131.5 nm post-testing, as shown in Figure 6 and Figure 7. In addition, the statistical analysis (independent t-test) indicated a significant difference (P < 0.05) in surface roughness before and after solubility testing of both tested materials, as shown in Table 2.

**Table 2:** The surface roughness values (Ra) of Omnichroma and Filtek™ Z250 XT specimens using AFM. * P < 0.05 was accepted as a significance level. * An independent t-test was performed. Ra1, calculated average between peaks and valleys on a surface before performing the test; Ra2, calculated average between peaks and valleys on a surface after performing the test AFM, atomic force microscopy; FL, Filtek; OM, Omnichroma

Specimen	R_a_1 (nm)	Average (nm)	R_a_2 (nm)	Average (nm)	*P-value
OM1	35.6874	17.03324	45.8817	33.1481	0.00821
OM2	3.94619		34.7281		
OM3	3.54613		18.5272		
OM4	18.0108		25.4375		
OM5	23.9757		41.166		
FL1	24.0495	18.3734	40.557	49.5171	0.019999
FL2	15.8139		19.9283		
FL3	14.2167		67.1182		
FL4	22.2408		46.8907		
FL5	15.5461		73.0913		

## Discussion

The current study compared the Wsp and Wsl values of a single-shade resin composite to those of a conventional resin composite in accordance with ISO 4049:2019 (Dentistry - Polymer-based Restorative Materials). ISO standard 4049:2019 for dental restorative resins specifies a maximum acceptable value of 40 μg/mm^3^ for Wsp and 7.5 μg/mm^3^ for Wsl. The statistical analysis failed to reject the null hypothesis. The findings revealed that the Wsl values of Omnichroma and Filtek™ Z250 XT did not exceed the upper limit of the acceptable value of the standard, and no significant difference was found between the tested groups of both materials.

The huge development of the nanotechnology industry has led to the production of smaller particle sizes and increasing filler volume in manufacturing resin composites in the dental field to promote mechanical and optical properties [15]. Omnichroma is the first resin-based composite restorative material that has the ability to match any tooth color and was introduced in 2019 by Tokuyama Dental. Tokuyama’s research suggests that the composite’s filler, consisting of 260 nm spherical particles, can improve light transmission and generate color parameters that better match the surrounding natural tooth structure [16]. This material covers a wide range of all VITA classical shades using a smart chromatic technology that is based on uniform spherical fillers (260 nm) [13]. It is produced with only specific, single-sized uniform spherical particles, which are developed with the Sol-Gel method as reported by the manufacturer. On the other hand, manufacturing conventional nano-hybrid resin composites involves crushing fillers until they reach the proper size of particles. Therefore, these uniform spherical filler particles are assumed to make single-shade resin composites less susceptible to being affected by the different surrounding conditions compared to conventional resin composites after prolonged immersion in water.

It has been reported that changes in pH and exposure to food and different drinks are among other factors that can significantly affect the surface integrity of resin-based composite restoration materials [17]. It is known that the quality of the surface of composite restorations is crucial to preserving their aesthetic appearance. Several factors can diminish the surface quality and therefore lead to an increase in surface roughness and changes in surface color [18]. Previous studies have claimed that rougher surfaces are more likely to accumulate plaque and induce bacterial colonization, which eventually may reduce the mechanical and optical properties of composite restorations [19]. In addition, these porosities may lead to mass loss (Wsl) and Wsp, which will lead to further surface roughness and discoloration [20].

The outcomes of this study showed that both materials are affected when exposed to water for a prolonged period of time, although the statistical analysis demonstrates no significant difference between both materials regarding Wsp and Wsl. However, the mean Wsp of Omnichroma specimens (9.05 µg/mm^3^) was less than the mean of Filtek™ Z250 XT specimens (10.18 µg/mm^3^). Furthermore, Wsl results demonstrated that the mean of Omnichroma specimens was -12.44 µg/mm^3^ and the mean of Filtek™ Z250 XT specimens was -9.05 µg/mm^3^, as shown in Table 1. These findings have not shown any mass loss (Wsl); this is mainly due to the Wsp property of resin composites. Therefore, this resulted in negative solubility values, as presented earlier. Considering the process of Wsp, it can be suggested that Omnichroma resin composite is less soluble than Filtek™ Z250 XT resin composite.

A previous study reported that the Wsp of Omnichroma resin composite was significantly higher than Essentia resin composite [21,22]. This can be linked to UDMA/TEGDMA as the main component of the Omnichroma matrix, which is more sensitive to pH change and its hydrophilic nature compared to the Essentia matrix, which is mainly based on BISEMA/TEGDMA. However, this study reported that the Wsl measurements of Essentia samples were significantly higher than Omnichroma samples [22]. This might be due to the infiltration of water molecules into the polymer network, resulting in uncured monomers diffusing out of the material structure and increasing the solubility. Another study has investigated the Wsp and Wsl of Omnichroma resin composite in three different conditions (distilled water, 5,000 thermocycles, and 10,000 thermocycles) [23]. The outcomes of all three groups showed that there was no significant difference in Wsp. However, the solubility results showed a considerable increase in Wsl between the tested groups. It has been reported that many factors can enhance the rate of solubility, such as pH and elevated temperature [24]. This could explain the reason for the increased Wsl of Omnichroma using the thermocycling method.

It has been proven that the Wsp and Wsl of resin composites can lead to mechanical degradation [4]. One of the most essential mechanical properties is the surface roughness of restorative materials [25]. A rough surface resin composite restoration can enhance plaque accumulation and have a negative effect on the durability and esthetic properties of the restorations [26,27]. Even though the Omnichroma nano-hybrid composite has a unique structure of uniformly spherical nanoparticles (260 nm) and the Filtek™ Z250 nano-hybrid composite is composed of a non-aggregated combination of silica, zirconia, and zirconia/silica cluster fillers ranging between 3 µm and 20 nm, the AFM images showed a noticeable increase in surface roughness after testing. The outcomes of the current study showed a higher surface roughness post-testing compared to pre-testing for Omnichroma and Filtek™ Z250 XT materials, as shown in Figure 4, Figure 5, Figure 6, and Figure 7. This indicates that even resin composites with smaller and uniform particles are subjected to degradation under certain conditions, such as being in the water for a prolonged period of time. However, the degree of the increased roughness as reported in the current study of both materials is still below the threshold level (Ra = 0.2 μm) for bacterial retention as reported in a previous study, as shown in Table 2 [28].

Another study evaluated the Wsl level and surface roughness and found that Essentia composite measures were greater than Omnichroma composite after thermocycling [29]. Therefore, this higher roughness and the uneven distribution of filler sizes in this type of resin-based composite could explain the reason for increasing the solubility values of Essentia composite compared to Omnichroma.

Aydin et al.’s study evaluated the surface roughness and found that there was no statistically significant difference between single and multi-shade resin-based composites. Also, it was stated that there was an effect of diamond finishing and polishing on surface roughness, which created less surface roughness on Filtek™ Z250 XT nano-hybrid resin composite [21]. A possible limitation of the current study was that the study specimens were not finished and polished as they were applied clinically, although this was not indicated by the ISO 4049:2019 standard for Wsp and Wsl testing methods. A polished surface may show more resistance to surrounding conditions, as reported in previous studies. Thus, it can be recommended to evaluate the Wsp and Wsl of resin composites with polished samples in order to anticipate their longevity in the oral cavity in future research studies.

However, considering the results of this study, the specimens of both materials did not influence their compliance with the acceptable measures of Wsp and Wsl as specified in the ISO 4049:2019 standard.

## Conclusions

Under the limitations of this study, it can be concluded that the Wsp and Wsl of Omnichroma (single-shade nano-hybrid composite) and Filtek™ Z250 XT (a conventional nano-hybrid composite) were within the acceptable range according to ISO 4049:2019, and there was no significant difference between the tested materials. In addition, the surface topography of Omnichroma specimens was less affected compared to Filtek™ Z250 XT specimens after performing the Wsp and Wsl tests, as per ISO 4049:2019, although both materials demonstrated significant differences in surface roughness. Therefore, understanding these factors helps in developing durable, reliable, and aesthetically pleasing composite materials, ensuring better clinical outcomes, and guiding future innovations in dental material science.
